# Concentration-Dependent Enhancement of Linear and Nonlinear Optical Properties in Hybrid Systems of Perylenediimide and Silver Nanoparticles

**DOI:** 10.3390/nano16050326

**Published:** 2026-03-05

**Authors:** Tarek Mohamed, Majed H. El-Motlak, Fatma Abdel Samad, Mohamed E. El-Khouly, Alaa Mahmoud

**Affiliations:** 1 Laser Institute for Research and Applications LIRA, Beni-Suef University, Beni-Suef 62511, Egypt; 2 Al Anbar Health Directorate, Training and Human Development Centre, Ramadi 31001, Al-Anbar, Iraq; 3 Nanoscience Program, Faculty of Basic and Applied Sciences, Egypt-Japan University of Science and Technology, New Borg El-Arab City 21934, Egypt

**Keywords:** nonlinear optical properties, femtosecond laser, Z-scan technique, perylene dye, laser ablation, laser induced fluorescence

## Abstract

The interaction between plasmonic nanoparticles and organic dye molecules plays an important role in varied photonic and optoelectronic applications. In this work, we systematically investigate the optical properties of a water-soluble perylenediimide derivative, N,N′-di(2-(trimethylammonium iodide) ethylene) perylenediimide (TAIPDI), in the presence of different concentrations of silver nanoparticles (AgNPs) under femtosecond (fs) laser excitation. The AgNPs were synthesized via the laser ablation technique. The influence of AgNP concentration on the linear, fluorescence, and nonlinear optical properties of the TAIPDI dye was explored through UV–visible absorption spectroscopy, fluorescence emission measurements, and open- and closed-aperture Z-scan techniques. The Ag NP–TAIPDI dye hybrid systems (Ag@TAIPDI nanocomposites) exhibited pronounced reverse saturable absorption and self-defocusing behavior, indicating a negative nonlinear refractive index. Both the nonlinear absorption coefficient and refractive index increased markedly with rising AgNP concentration, leading to a significant enhancement in the third-order nonlinear susceptibility. Fluorescence studies further revealed a concentration-dependent emission enhancement due to metal-enhanced fluorescence arising from surface plasmon resonance-induced local field amplification. The Ag@TAIPDI nanocomposites also demonstrated strong optical limiting performance, with the limiting threshold decreasing as the AgNP concentration increased. These findings highlight the synergistic role of plasmon–exciton coupling and thermal lensing in enhancing the nonlinear response of such nanocomposites. The results establish AgNPs–TAIPDI dye hybrid systems as promising materials for all-optical switching, optical limiting, and photonic device applications.

## 1. Introduction

The rapid advancement of modern photonics and optoelectronics has created a growing demand for novel nonlinear optical (NLO) materials with strong and ultrafast optical responses [1,2,3]. Organic dyes have attracted considerable attention due to their exceptionally large and rapid nonlinear responses, simple fabrication methods, low cost, and structural tenability [4,5,6,7]. They are used as potential candidates in various optoelectronic applications, including optical limiting, optical data storage, photonic switching systems, and optical communications [8,9]. To characterize such materials, the single-beam Z-scan technique, introduced by Sheik-Bahae et al. [10], has become one of the most reliable and widely used methods for determining both the sign and magnitude of third-order NLO susceptibility.

Recently, hybrid systems composed of organic dyes and plasmonic nanoparticles (NPs) have emerged as highly promising candidates for NLO applications [11]. Dye-doped metal NPs are a distinct class of nanostructured materials that integrate the strong electronic transitions of organic dyes with the plasmonic properties of metal NPs such as silver and gold. These dye–metal nanocomposites exhibit enhanced light–matter interactions, tunable optical responses, and remarkable potential for use in sensing, imaging, photonic devices, and NLO systems [12,13,14]. The distinctive optical behavior of metallic NPs originates from localized surface plasmon resonances (LSPRs), which generate intense electromagnetic fields near the nanoparticle surface and can strongly couple to the electronic transitions of nearby dye molecules [15,16]. When organic dye molecules are doped into or adsorbed onto metal NPs, plasmon–exciton coupling can occur, resulting in novel and often enhanced optical features, such as fluorescence amplification, spectral shifts, and energy transfer effects [17]. Moreover, dye–metal NP hybrids have been extensively investigated for third-order NLO phenomena such as two-photon absorption, third-harmonic generation, and optical limiting [18]. The synergistic combination of the local field enhancement from the metallic core and the large intrinsic nonlinear susceptibility of the dye molecules results in significantly amplified nonlinear responses, making these systems promising for optical switching, modulation, and laser protection applications.

Noble metal NPs exhibit remarkable NLO properties, making them attractive candidates for the development of next-generation nanoscale photonic and optoelectronic devices [19]. In particular, silver nanoparticles (AgNPs) display extremely weak intrinsic photoluminescence, with nonradiative relaxation serving as the dominant deactivation pathway under optical excitation [20]. However, the strong localization of electromagnetic fields near the nanoparticle surface, caused by localized surface plasmon resonance (LSPR), can significantly enhance the fluorescence intensity of nearby fluorophores by several orders of magnitude [21,22]. AgNPs can be synthesized using the pulsed laser ablation (PLA) technique, which is a simple, clean, and controllable top-down method that employs a pulsed Nd: YAG laser (532 nm) to generate colloidal NPs. The size, morphology, and stability of the resulting NPs can be tuned by adjusting parameters such as laser fluence, wavelength, pulse width, and the dielectric medium [23,24].

Organic dyes, which exhibit strong intramolecular charge transfer between donor and acceptor moieties, have been widely employed in various optoelectronic and photonic applications, including organic light-emitting diodes, chemical sensors, and dye-sensitized solar cells. Their physicochemical behaviour, such as dielectric constant, transition dipole moment, and fluorescence quantum yield, is highly sensitive to the surrounding environment, including solvent polarity [25,26]. Among the numerous organic dyes, perylenediimide (PDI) derivatives have attracted significant attention owing to their high molar absorptivity in the visible region, near-unity fluorescence quantum yield, exceptional photochemical stability, low electron affinity (EA ≈ −3.9 eV), and excellent chemical robustness [27,28,29]. These unique features have enabled PDIs to serve as functional materials in a wide range of devices, such as organic field-effect transistors [30], dye lasers [27,31], organic photovoltaic cells [32,33], and optical limiters [34,35].

In the present study, we investigate the influence of AgNPs on the linear and NLO behavior of TAIPDI dye under femtosecond (fs) laser excitation at 800 nm. The third-order NLO parameters, including the nonlinear absorption (*β*), nonlinear refractive index (*n*_2_), and third-order susceptibility (χ^(3)^), were experimentally determined using open- and closed-aperture Z-scan measurements. A pronounced enhancement in the NLO response and optical limiting behavior was observed with increasing Ag NP concentration, attributed to plasmon-induced local field amplification and efficient energy transfer between TAIPDI molecules and AgNPs. These results highlight the potential of Ag@TAIPDI hybrid systems as promising materials for advanced photonic and optical limiting applications.

## 2. Experimental Details

### 2.1. Synthesis of AgNPs Colloids

**Figure 1:**
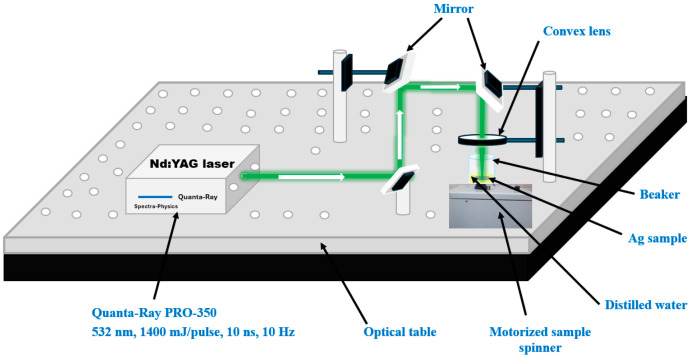
Laser ablation setup for preparing AgNPs colloids via a 532 nm Nd: YAG laser.

Figure 1 illustrates the experimental setup used for the synthesis of AgNPs colloids via the pulsed laser ablation PLA method. A second-harmonic Nd: YAG laser system (Spectra Physics–Quanta-Ray PRO 350, Spectra-Physics Inc., Milpitas, CA, USA.) operating at a wavelength of 532 nm, with a pulse duration of 10 ns and a repetition rate of 10 Hz, was employed. The laser produced a maximum pulse energy of 1500 mJ and a Gaussian beam profile (TEM_00_ mode). A high-purity silver target (>99%) was submerged in 10 mL of distilled water inside a beaker and irradiated for 30 min. Before ablation, the silver surface was mechanically polished to remove any oxide layer caused by air exposure and ultrasonically cleaned in ethanol and deionized water for 30 min to eliminate organic contaminants. A convex lens with a 10 cm focal length was used to focus the laser beam onto the target surface. Under these conditions, the laser operated at an average power of 700 mW, which corresponds to a laser fluence of 2.91 J/cm^2^. The focal spot diameter was estimated to be approximately 1.75 mm, as determined using the knife-edge method. To ensure uniform ablation and minimize particle aggregation, the beaker was rotated at 177 rpm using a motorized stage. This method provides a clean and controllable synthesis route, producing stable, spherical AgNPs without requiring chemical precursors or surfactants.

### 2.2. Preparation of TAIPDI Dye and Ag@TAIPDI Nanocomposites

The stock solution of perylenediimide was prepared as reported in Refs. [35,36]. A dye solution with a final concentration of 7.2 × 10^−6^ M was obtained by serial dilution using distilled water, according to the standard relation:

(1) *C*_1_*V*_1_ = *C*_2_*V*_2_,

where *C*_1_ is the greater (stock) concentration, *C*_2_ is the lowest (needed) concentration, *V*_1_ is the volume of the greater concentration required to add, and *V*_2_ is the volume of the lowest (needed) concentration. Separately, AgNPs synthesized via PLA were diluted with distilled water to obtain four concentrations: 2.3, 3.37, 4.5, and 5 mg/L. These AgNPs colloids were then mixed with the TAIPDI dye solution to form Ag@TAIPDI nanocomposites. The mixtures were stirred magnetically for 30 min to ensure homogeneous dispersion and uniform nanoparticle–dye interaction.

### 2.3. Fluorescence Measurements

**Figure 2:**
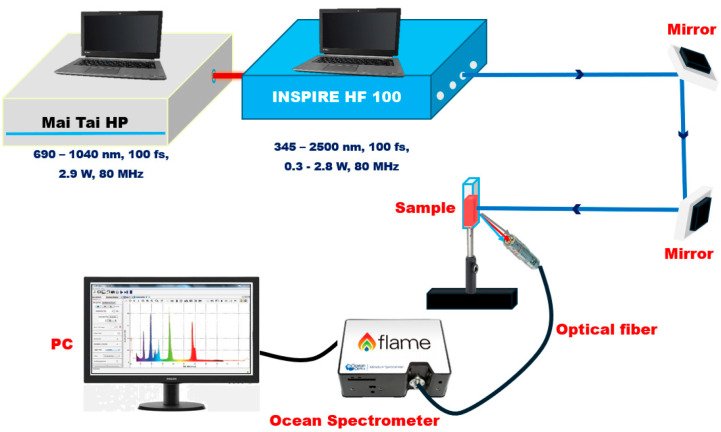
Laser-Induced Fluorescence (LIF) experimental setup for analyzing the fluorescence characteristics of the TAIPDI and Ag@TAIPDI nanocomposite samples.

The fluorescence characteristics of the TAIPDI and Ag@TAIPDI nanocomposite samples were examined to evaluate the plasmonic enhancement of emission. The laser-induced fluorescence (LIF) setup, schematically illustrated in Figure 2, utilized a fs laser system (Spectra-Physics INSPIRE HF100, Spectra-Physics Inc., Milpitas, CA, USA), pumped by a Ti: sapphire mode-locked MAI TAI HP laser (Spectra-Physics, Spectra-Physics Inc., Milpitas, CA, USA), with 1.5–2.9 W average power, 80 MHz repetition rate, and wavelength ranging from 690 to 1040 nm. The TAIPDI and Ag@TAIPDI nanocomposite samples were placed in a 1 mm path-length micro quartz cuvette (chamber volume ≈ 350 μL), which exhibited no intrinsic emission within the spectral range of interest (200–2500 nm). The excitation wavelength was set at 420 nm, corresponding to the primary absorption band of the TAIPDI dye. The laser beam was carefully aligned and focused onto the cuvette through highly reflective mirrors to ensure optimal beam stability and uniform excitation. The emitted fluorescence was collected using a convex lens and coupled via an optical fiber to a spectrometer (Ocean Optics FLAME–S–XR1, 3500 Quadrangle Blvd. Orlando, FL, USA; spectral range 200–1025 nm, resolution 1.69 nm FWHM). The recorded spectra were displayed and stored on a PC monitor for subsequent analysis.

### 2.4. Z-Scan Setup

**Figure 3:**
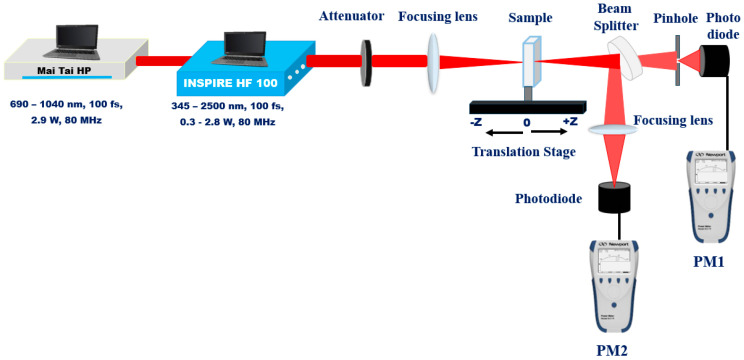
Z-scan experimental setup.

The NLO properties of the samples were measured using the single-beam Z-scan technique, as illustrated schematically in Figure 3 [37,38]. The same fs laser source (INSPIRE HF100, pumped by a MAI TAI HP Ti: sapphire laser) was employed at a wavelength of 800 nm. The laser beam, characterized by a Gaussian spatial profile (TEM00 mode) and a beam quality factor of M^2^ < 1.1, was tightly focused using a 5 cm focal length convex lens. The samples were placed in 1 mm path-length quartz cuvettes mounted on a micrometer translation stage to enable precise scanning around the focal point of the beam. The transmitted intensity was recorded using a Newport 843R power meter, Spectra-Physics Inc., Milpitas, CA, USA (PM) as a function of the sample position (z) relative to the focus. For the closed-aperture (CA) measurements (aperture set to *S* = 0.3), both the sign and magnitude of the nonlinear refractive index (*n*_2_) were determined using PM1. For the open-aperture (OA) configuration (*S* = 1; fully open), the nonlinear absorption coefficient (*β*) was extracted from the transmittance variation arising from intensity-dependent absorption, using PM2. The experimental Z-scan data were fitted with standard theoretical models under the thin-sample approximation to extract the relevant NLO parameters.

## 3. Results and Discussions

### 3.1. Characterization of AgNPs

**Figure 4:**
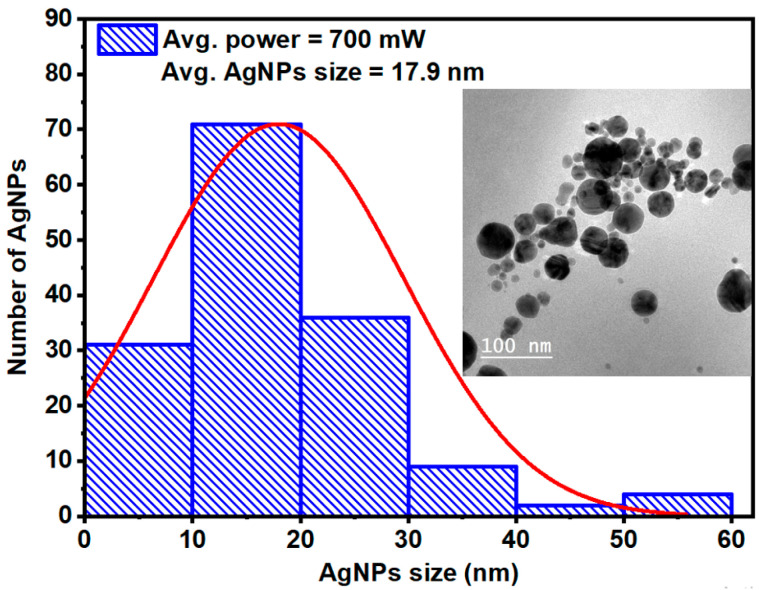
Size distribution histogram for AgNPs. The TEM images of the generated spherical AgNPs are also included in the inset figure. The red curve represents the Gaussian fit to the AgNP average size distribution.

The concentration of the synthesized AgNPs colloids was determined using inductively coupled plasma optical emission spectroscopy (ICP-OES, Agilent 5100 Synchronous Vertical Dual View with VGA 77 vapor generation accessory). Under an average laser power of 700 mW, an exposure time of 30 min, and a wavelength of 532 nm, the AgNP concentration was measured to be approximately 10 ± 1 mg/L. The morphology and size distribution of the AgNPs were characterized using high-resolution transmission electron microscopy (HR-TEM, JEM-2100, JEOL, Akishima, Tokyo, Japan) operated at 200 kV. Particle diameters were measured from multiple regions of the TEM images using ImageJ software, ImageJ 1.x, which was also used to generate histograms and calculate the average particle size. The resulting size distribution, with TEM images inset, shown in Figure 4, confirms that the Ag NPs are predominantly spherical with an average diameter of 17.9 ± 1.8 nm.

### 3.2. Linear Optical Properties of Ag@TAIPDI Nanocomposites

#### 3.2.1. UV–Visible Extinction Analysis

**Figure 5:**
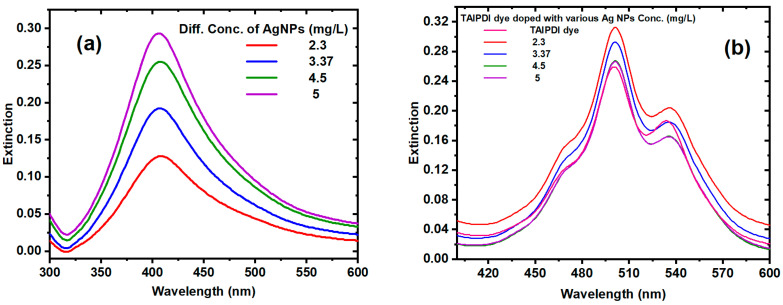
Extinction spectra of (**a**) AgNPs, and (**b**) TAIPDI dye doped with AgNPs (Ag@TAIPDI nanocomposites) at different AgNP concentrations.

The optical extinction spectra of AgNPs and Ag@TAIPDI nanocomposites were recorded using a UV–visible spectrophotometer (Model C-7200) in the wavelength range of 200–1100 nm. Figure 5 shows the extinction spectra of (a) AgNPs synthesized via laser ablation at 700 mW average power and an exposure time of 30 min, and (b) Ag@TAIPDI nanocomposites prepared with varying AgNP concentrations (2.3, 3.37, 4.5, and 5 mg/L). For the AgNPs colloid (Figure 5a), a distinct surface plasmon resonance (SPR) band appears at 406 nm, indicating the formation of spherical nanoparticles with uniform size distribution. When AgNPs were incorporated into the TAIPDI matrix (Figure 5b), the extinction characteristics of the dye were notably altered due to LSPR coupling. Perylenediimide (PDI) dyes exhibit strong and well-defined π–π* absorption bands in the visible region; however, upon interaction with AgNPs, these bands show variations in intensity and slight changes in shape, depending on nanoparticle concentration and dispersion.

**Figure 6:**
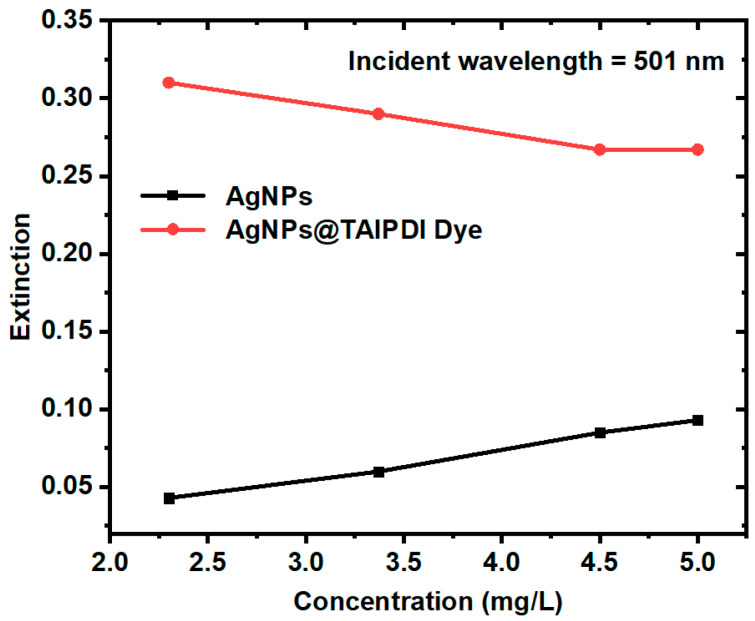
Extinction of AgNPs and Ag@TAIPDI nanocomposites as a function of AgNP concentration.

Accordingly, the linear optical properties of Ag@TAIPDI nanocomposites depend non-monotonically on the AgNP concentration. As shown in Figure 6, the extinction of both AgNPs and Ag@TAIPDI nanocomposites. The Ag@TAIPDI samples exhibit significantly higher extinction than the bare AgNPs, indicating the pronounced effect of LSPR on the TAIPDI dye. The figure also indicates that the extinction of Ag@TAIPDI nanocomposites slightly decreases at higher concentrations, which can be explained as follows. At low AgNP concentrations, the nanoparticles are well dispersed and efficiently enhance the local electromagnetic field through LSPR, leading to increased extinction and fluorescence intensity of nearby TAIPDI dye molecules through metal-enhanced extinction and fluorescence effects. As the nanoparticle concentration increases, inter-particle interactions and aggregation become significant, resulting in plasmon–plasmon coupling and broadening of the plasmon resonance. This reduces the effectiveness of near-field enhancement at the dye extinction wavelength. In addition, higher nanoparticle concentrations introduce increased optical scattering and partial shielding of the dye molecules, which reduces the effective light intensity reaching the dye. Furthermore, since only dye molecules within a limited near-field distance from the nanoparticle surface experience plasmonic enhancement, the system reaches a saturation regime, beyond which additional nanoparticles do not contribute to further extinction enhancement. These combined effects lead to a net decrease in the measured extinction of the dye–nanoparticle mixture at higher nanoparticle concentrations [39,40].

#### 3.2.2. Energy Bandgap and Linear Refractive Index

The energy band gap (*E_g_*) of the Ag@TAIPDI nanocomposite dye solutions is calculated from the optical extinction data using Tauc’s plot equation [41].

**Figure 7:**
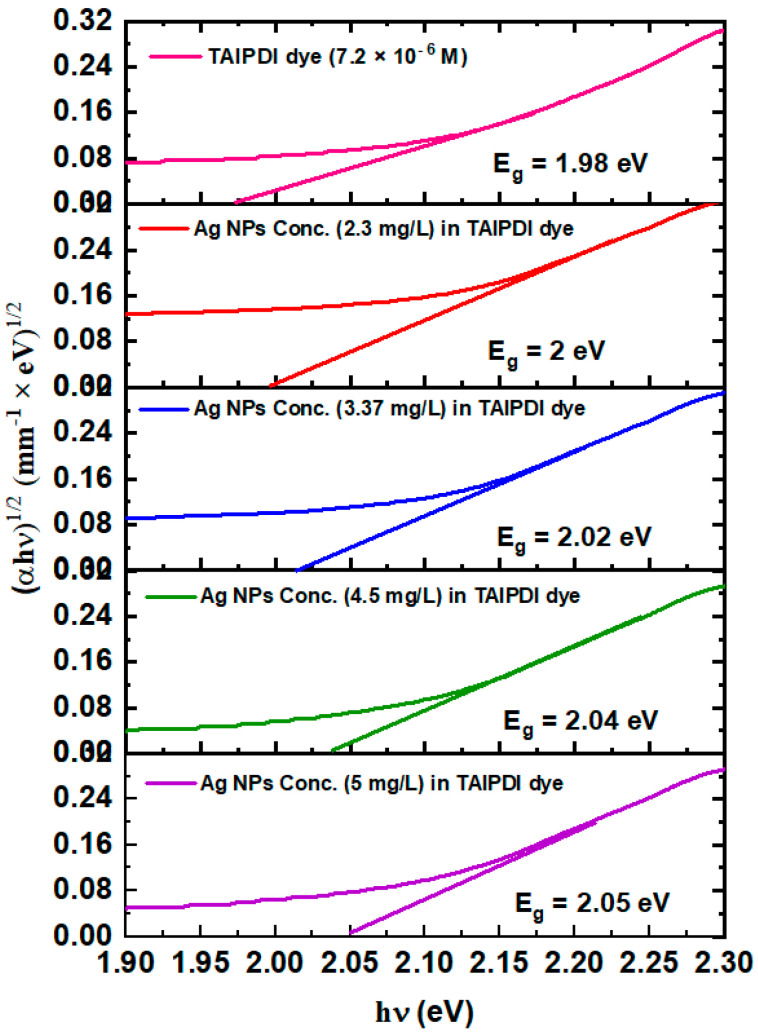
Energy band gaps estimated from Tauc’s plots of Ag@TAIPDI nanocomposites at various AgNPs colloidal concentrations in water.

(2) (*αhν*)^1/2^ = *a*(*hν* − *E_g_*)

where α is the linear extinction coefficient (calculated from extinction *A* and sample thickness *t* as α = 2.303 *A*/*t* [42]), *hν* is the photon energy, and *a* is a constant. Figure 7 depicts the *E_g_* values for the Ag@TAIPDI dye solutions obtained from the (*αhν*)^1/2^ versus *hν* plots [43]. The *E_g_* values of the Ag@TAIPDI dye can be deduced by extrapolating the linear part of the plot to *αhν* = 0. The extracted bandgap values (Figure 7) show a gradual increase from 2.00 to 2.05 eV as the AgNP concentration increases from 2.3 to 5 mg/L, indicating a concentration-dependent modulation of the electronic structure. The transmission results were used to calculate the linear refractive index (n_0_) via the Swanepoel formalism [44].

(3) $$n_{o}=\frac{1}{T}+{\left(\frac{1}{T^{2}}-1\right)}^{1/2}$$

where *T* represents the transmittance of the Ag@TAIPDI nanocomposite dye sample. The refractive index of the Ag@TAIPDI dye solution varied from 1.23 to 1.12 as the AgNP concentration increased from 2.3 mg/L to 5 mg/L, demonstrating a clear concentration-dependent optical behavior of the composite system.

#### 3.2.3. Effect of AgNPs on the Fluorescence Properties of TAIPDI Dye

**Figure 8:**
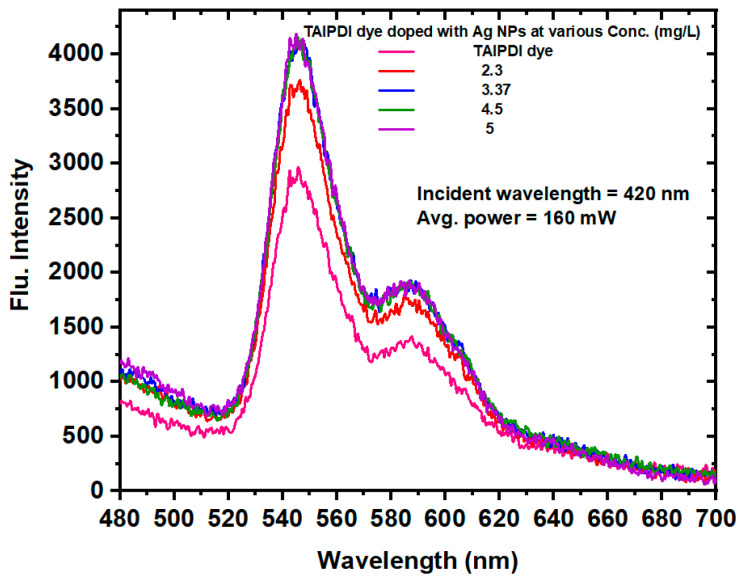
Fluorescence spectra obtained by 420 nm laser excitation of TAIPDI dye doped with AgNPs obtained using Laser-Induced Fluorescence.

The fluorescence emission spectra of the TAIPDI dye and Ag@TAIPDI nanocomposites were recorded to evaluate the influence of AgNPs on the photophysical behavior of the dye. Figure 8 presents the Laser-Induced Fluorescence (LIF) spectra obtained under excitation at 420 nm and a constant incident laser power of 160 mW. The concentration of AgNPs in the dye solution was systematically varied from 2.3 to 5 mg/L. A distinct enhancement in fluorescence intensity was observed with increasing AgNP concentration. This effect is attributed to the LSPR of the AgNPs, which generates strong electromagnetic fields near the nanoparticle surface when excited by light. When the excitation wavelength of the dye overlaps with the LSPR band of the AgNPs, dipole–plasmon coupling occurs, leading to efficient energy transfer between the NPs and the dye molecules. The resulting local field amplification enhances both the extinction and radiative decay rates of the fluorophores, thereby increasing emission intensity, a phenomenon commonly referred to as metal-enhanced fluorescence (MEF) [45,46,47]. The MEF has emerged as a promising approach that significantly improves fluorescence efficiency, offering great potential for advancing biomedical imaging, chemical sensing, and organic optoelectronic applications. Furthermore, the presence of AgNPs can suppress dye aggregation and nonradiative decay pathways, contributing to the observed fluorescence amplification. The degree of enhancement depends on the nanoparticle concentration, interparticle spacing, and distance between the dye molecules and nanoparticle surfaces. Excessive nanoparticle loading, however, may lead to fluorescence quenching due to energy transfer to the metal surface. The overall results confirm that the plasmonic coupling between TAIPDI molecules and AgNPs plays a crucial role in modulating the emission efficiency of the dye, providing a pathway for improved photonic and sensing applications. It is worth noting that the decreasing absorption at higher AgNP concentration observed in Figure 5b does not contradict the monotonic increase in fluorescence intensity shown in Figure 8, since they result from two different phenomena. Extinction reflects the number of well-dispersed nanoparticles and can decrease at higher concentrations due to aggregation, scattering, or SPR broadening/shifting [39,48]. In contrast, fluorescence depends on excitation efficiency and radiative decay and can increase via MEF if nanoparticles remain dispersed and quenching is minimal. At certain concentrations, MEF dominates, yielding a monotonic fluorescence increase [40,49], while extinction may still decrease due to morphological changes. It is worth noting that the fluorescence lifetime measurements were not performed in this study; therefore, the enhancement mechanism is interpreted based on steady-state photoluminescence results. MEF can arise from increased radiative decay rates due to plasmon–exciton coupling and from modifications of the local density of optical states (LDOS) near metallic nanoparticles.

**Figure 9:**
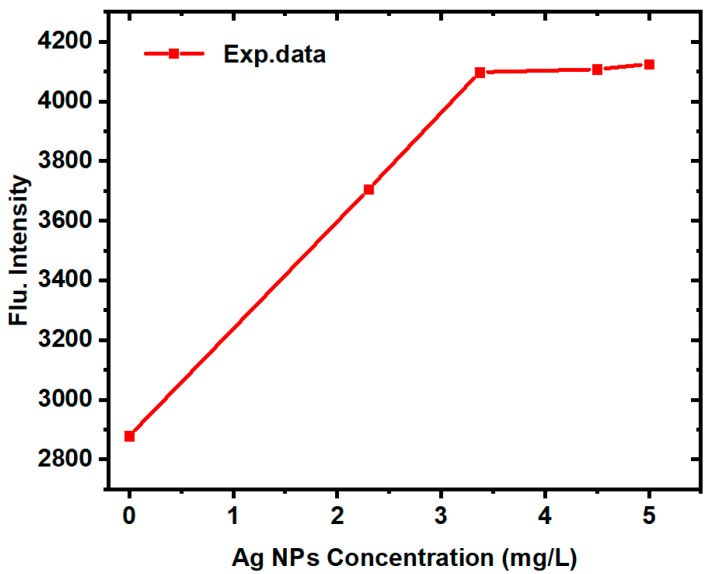
Fluorescence intensity of Ag@TAIPDI nanocomposites as a function of AgNP concentration, showing enhancement at low concentrations and saturation at higher concentrations.

The Fluorescence intensity of Ag@TAIPDI nanocomposites as a function of AgNP concentration is shown in Figure 9. The figure shows that at low AgNP concentrations (0–3 mg/L), the fluorescence intensity increases sharply due to efficient metal-enhanced fluorescence caused by the LSPR of well-dispersed nanoparticles. Beyond ~3 mg/L, plasmon–plasmon coupling, increased scattering, partial aggregation, and reduced effective dye–metal separation control lead to saturation. No strong quenching was observed in the investigated range; instead, enhancement approaches a plateau due to near-field saturation, consistent with the decrease in absorption efficiency discussed earlier. These competing effects explain the observed manner in the extinction and fluorescence spectra. The interaction between the excitonic transitions of TAIPDI molecules and the plasmonic fields of AgNPs results in enhanced light absorption, particularly near the plasmon resonance wavelength of silver. This enhancement is attributed to the local amplification of the electromagnetic field around the AgNPs, which induces stronger electronic excitation in nearby dye molecules [50,51]. Additionally, doping with AgNPs suppresses dye aggregation, thereby improving the clarity and stability of the absorption spectrum. These findings indicate a notable interaction between AgNPs and TAIPDI, which could be of interest for further studies on optoelectronic and nonlinear optical properties.

### 3.3. Nonlinear Optical Properties of Ag@TAIPDI Nanocomposites

#### 3.3.1. Open-Aperture (OA) Z-Scan: Nonlinear Absorption

**Figure 10:**
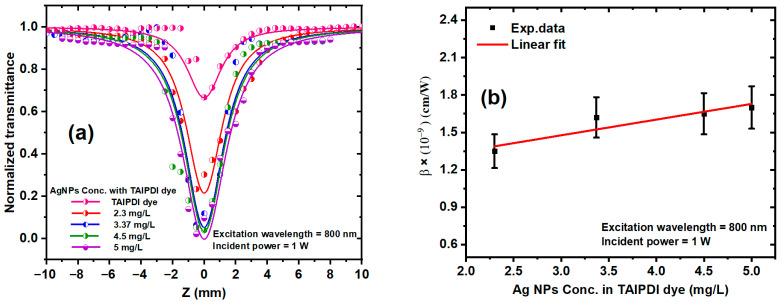
(**a**) OA Z-scan transmission of TAIPDI dye and Ag@TAIPDI nanocomposites at a dye concentration of 7.2 × 10^−6^ M at an excitation wavelength of 800 nm, 1 W incident power, and different AgNP concentrations in water. Dots are the experimental data, whereas the solid curves are the theoretical fits. (**b**) The AgNP concentration as a function of the obtained values of β for the Ag@TAIPDI nanocomposites. Dots are experimental data, while the solid line is the linear fit. Error bars correspond to the confidence interval in the measured values.

The nonlinear absorption (NLA) behavior of the TAIPDI dye and Ag@TAIPDI nanocomposites was investigated using the OA Z-scan configuration. Figure 10a displays the normalized transmittance curves measured at an excitation wavelength of 800 nm and a constant incident power of 1 W. The TAIPDI concentration was fixed at 7.2 × 10^−6^ M, while the AgNP concentration was varied from 2.3 to 5 mg/L. The energy band gap of TAIPDI in water was determined to be 1.98 eV; therefore, two-photon absorption (2PA) is expected to occur at a photon energy of about 1.55 eV, corresponding to a wavelength of 800 nm. All samples exhibited reverse saturable absorption (RSA) characteristics, evidenced by symmetric transmittance minima (valley) at the focal point (z = 0). The RSA is an NLO phenomenon in which absorption increases with incident light intensity because the excited-state absorption cross section exceeds that of the ground state. As the excitation intensity rises, a higher population of molecules is promoted to excited states, leading to enhanced absorption and reduced transmission. This indicates that the absorption increases with laser intensity, which can be attributed predominantly to effective 2PA, with a possible contribution from excited-state absorption (ESA) processes mediated by plasmon-enhanced excited-state population. The figure also reveals that the normalized transmittance (TOA) reduces when the AgNP concentration increases in TAIPDI dye. Typically, the NLA behavior of a material under high laser intensity (I) is influenced by the incident intensity and can be described by the relation

(4) $$\alpha \left(I\right)=\alpha +\beta I$$

where *β* denotes the 2PA coefficient. To evaluate the NLA coefficient, the experimental OA Z-scan data were fitted using the theoretical NLO absorption model expressed as follows [6,35]:

(5) $$T{\left(z\right)}_{OA}=1-\frac{\beta I_{o}L_{eff}}{2\sqrt{2}\left[1+{\left(z/z_{o}\right)}^{2}\right]}$$

where $I_{o}=2P/\pi \omega_{o}^{2}$ represents the peak intensity at the focal point (z = 0), and $z_{o}=n_{o}\pi \omega_{o}^{2}/\lambda$ is the Rayleigh length. Here, *P* is the peak power, *ω_o_* is the beam waist at the focus, and λ is the excitation wavelength. The effective length of the sample, *L_eff_*, is calculated as $L_{eff}=\left[1-exp(-\alpha L)\right]/\alpha$, where *L* denotes the actual thickness of the sample.

The experimental data in Figure 10a are fitted using Equation (5) to obtain the value of β for different AgNP concentrations. The extracted NLA coefficients *β* are summarized in Figure 10b. A clear increase in *β* with AgNP concentration was observed, confirming that incorporation of AgNPs enhances the NLA response of the dye. This enhancement arises from two synergistic effects: (i) the reduction of the optical bandgap caused by nanoparticle incorporation, which facilitates multi-photon transitions, and (ii) local field enhancement associated with the LSPR of AgNPs, which increases the effective optical intensity experienced by the dye molecules [52]. These findings demonstrate that plasmonic coupling strongly amplifies the NLA process in the hybrid system. It is worth noting that the pronounced enhancement of the third-order nonlinear susceptibility |χ^(3)^| observed in the Ag@TAIPDI nanocomposites cannot be attributed to the intrinsic nonlinear optical response of AgNPs alone. The only silver nanoparticles dispersed in aqueous media exhibit relatively modest third-order nonlinear responses [53]. Consequently, the observed enhancement in the nonlinear optical properties after the incorporation of the TAIPDI dye can be attributed mainly to synergistic plasmon–exciton coupling between the localized surface plasmon resonance of AgNPs and the electronic transitions of the TAIPDI molecules. This plasmon-induced local field amplification effectively increases the optical intensity experienced by the dye, leading to strengthened absorptive and refractive nonlinearities in the hybrid system.

#### 3.3.2. Closed-Aperture (CA) Z-Scan: Nonlinear Refraction

CA Z-scan measurements were performed to evaluate the nonlinear refractive index (*n*_2_) of the samples under the same excitation conditions (800 nm, 1 W) using 100 fs laser pulses at an 80 MHz repetition rate. It is worth noting that the interval between successive pulses (12.5 ns) is considerably shorter than the thermal characteristic time $t_{c}=\omega_{o}^{2}/4D$, which is typically ≥40 µs for liquids, where *D* is the thermal diffusion coefficient. Because the sample cannot return to thermal equilibrium between pulses, heat accumulates, leading to a non-uniform temperature distribution that modifies the spatial refractive-index profile. Because the sample cannot return to thermal equilibrium between pulses, heat accumulates, leading to a non-uniform temperature distribution that modifies the spatial refractive-index profile. This cumulative heating distorts the CA Z-scan signal and may influence the extracted nonlinear refractive (NLR) index. In the case of thermal nonlinearity and steady-state equilibrium, the on-axis variation of the NLR index (Δ*n*) can be expressed as

(6) $$\Delta n=\frac{dn}{dT}\frac{I\alpha \omega_{o}^{2}}{4k}$$

where *k* is the thermal conductivity and dn/dT is the thermo-optic coefficient of the sample. When electrons absorb one or two photons of identical energy, they are promoted from the ground to the excited state, a population-redistribution process that largely governs the refractive nonlinearity of Ag@TAIPDI nanocomposites in the fs regime. The number of laser pulses that contribute to cumulative thermal lensing during the scan depends on the repetition rate and the position of the sample and is described by

(7) $$\frac{1}{f(Z)}=\frac{aLE_{p}F_{l}^{3/2}}{\omega {\left(Z\right)}^{2}}\left[1-\frac{1}{\sqrt{N_{p}}}\right]$$

where *a* is the fitting parameter, *a* = α (d*n*/d*T*)/2κ (π3*D*)^1/2^, $\omega \left(z\right)$ is the laser beam radius at the sample, *F_l_* is the repetition rate, *E_p_* is the pulse energy, and *N_p_* represents the number of incident laser pulses on the sample during the scan. Since *N_p_* = *t* × *F_l_*, where *t* is the exposure time for each TAIPDI sample in this work (*t* ≈ 3 min, *F_l_* = 80 × 10^6^ s^−1^) was irradiated by roughly 1.4 × 10^9^ laser pulses during each scan. For *f* ≥ z_0_, the normalized CA transmittance variations (ΔT_CA_) depend on the focal length of the induced thermal lens according to

(8) $$\Delta T_{CA}=1+\frac{2Z}{f(Z)}$$

**Figure 11:**
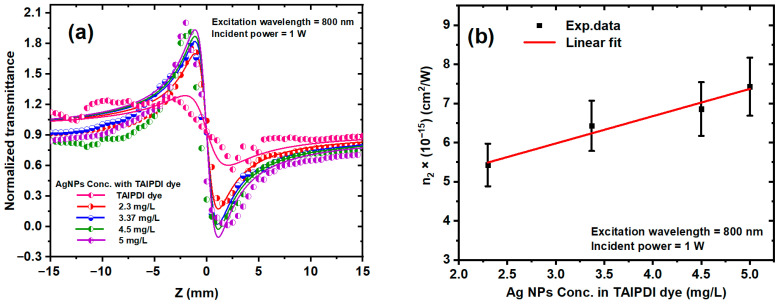
(**a**) CA Z-scan transmission for TAIPDI dye and Ag@TAIPDI nanocomposites at an excitation wavelength of 800 nm and at various AgNP concentrations. The symbols represent the experimental data, and the solid curves are the fits obtained utilizing Equations (7) and (8). (**b**) Relationship between the AgNP concentration and measured *n*_2_ for Ag@TAIPDI nanocomposites. The dots refer to experimental data, whereas the solid lines indicate linear fits.

Figure 11a shows the CA transmittance data of Ag@TAIPDI nanocomposites at different AgNP concentrations, keeping the dye concentration constant at 7.2 × 10^−6^ M. The figure exhibits a pre-focal peak followed by a post-focal valley, indicating a negative NLR index *n*_2_ (self-defocusing behavior). This self-defocusing effect arises from thermally induced refractive-index gradients that cause the beam to diverge inside the sample. Materials exhibiting negative *n*_2_ are of interest for optical limiting [54], all-optical switching [55], NLO devices [56], and soliton generation and propagation [57]. In this experiment, the aperture was adjusted to transmit 30% of the total light in the open-aperture configuration. The experimental data were fitted theoretically using Equations (7) and (8) to extract the thermal-lens parameters. At the focal position, the nonlinear phase shift (Δφ) as a function of the thermal focal length is given by [58]

(9) $$\Delta \varphi =\frac{z_{o}}{2f(0)}$$

where *f*(0) represents the focal length of the induced thermal lens when the sample is at z = 0. The dye solutions were contained in 1 mm path-length quartz cuvettes satisfying the thin-sample condition (z_o_ > *L*). Once Δφ is determined, the NLR index *n*_2_ can be evaluated as

(10) $$n_{2}=\frac{\lambda \omega_{o}^{2}\Delta \varphi }{2PL_{eff}}$$

Figure 11b displays the variation of *n*_2_ with AgNP concentration, obtained from Equations (7)–(10). The results reveal a clear increase in *n*_2_ of the Ag@TAIPDI nanocomposite samples as the AgNP concentration rises, indicating that the incorporation of AgNPs into the dye solution induces strong local-field effects associated with surface-plasmon resonance. These NPs act as efficient scattering centers, intensifying the interaction between the incident optical field and the TAIPDI molecules and thus enhancing the NLR response. It is worth noting that the n_2_ originates from the combined influence of thermal and electronic effects. The electronic contribution, known as the Kerr effect, arises from the instantaneous polarization of the electron cloud in response to an intense applied electromagnetic field.

#### 3.3.3. Third-Order Nonlinear Susceptibility (χ^(3)^)

The real and imaginary components of the third-order nonlinear susceptibility $\chi^{\left(3\right)}$ were calculated from the experimentally determined *n*_2_ and *β* values, respectively, using the relations [59]:

(11) $$Re\left[\chi^{\left(3\right)}\right]=\frac{n_{o}}{3\pi }n_{2},Im\left[\chi^{\left(3\right)}\right](esu)=\frac{{10^{-7}c\lambda n}_{o}}{96\pi^{2}}\beta$$

where *n_o_* is the linear refractive index, *c* is the speed of light. The total magnitude of the susceptibility is given by [6]

(12) $$\left|\chi^{\left(3\right)}\right|=\sqrt{\left({Re\left[\chi^{\left(3\right)}\right]}^{2}+{Im\left[\chi^{\left(3\right)}\right]}^{2}\right)}$$

The absolute values of $\chi^{\left(3\right)}$ are determined at different AgNP concentrations and summarized in Table 1. The calculated absolute values of $\chi^{\left(3\right)}$ increase markedly with AgNP concentration, demonstrating the concentration-dependent enhancement of third-order nonlinear polarization. The highest $\chi^{\left(3\right)}$ was obtained for the composite containing 2.3 mg/L of AgNPs, corresponding to approximately a threefold increase relative to the pure TAIPDI solution. This improvement reflects the strong electromagnetic coupling between the dye molecules and the plasmonic NPs, which effectively magnifies the local optical field and promotes NLO interactions.

### 3.4. Optical Limiting Effect of Ag@TAIPDI Nanocomposites AgNPs

**Figure 12:**
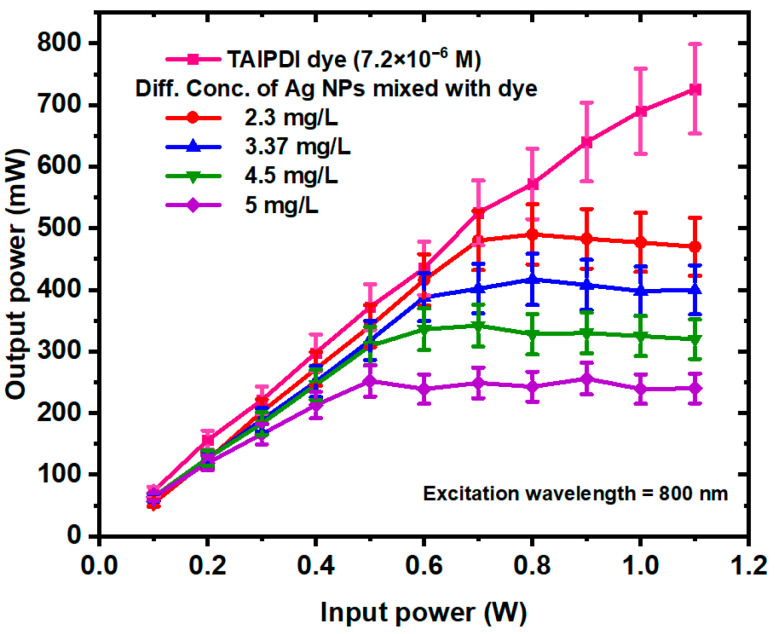
Optical limiting behavior of TAIPDI and Ag@TAIPDI nanocomposites at an 800 nm excitation wavelength, 1 W incident power, and at various AgNP concentrations (2.3–5 mg/L). The sample was kept fixed at the focus, and the aperture was fully open.

The optical limiting (OL) performance of the TAIPDI and Ag@TAIPDI nanocomposites was investigated using the same fs laser setup employed for the Z-scan measurements. The samples were placed at the focal point of the lens, and the transmitted power was recorded as a function of the incident laser power at a wavelength of 800 nm, which lies outside the primary absorption band of TAIPDI (470–540 nm). The resulting OL curves for different AgNP concentrations (2.3–5 mg/L) are presented in Figure 12. At low input powers, the output power increases linearly with the input, indicating a linear transmission regime. However, beyond a certain threshold, the transmission begins to saturate, demonstrating a clear OL response. The limiting behavior becomes more pronounced with increasing AgNP concentration, reflecting a reduction in the OL threshold from approximately 700 mW for the lowest nanoparticle concentration to about 500 mW for the highest. The observed OL behavior arises primarily from RSA and 2PA processes, as identified from the OA Z-scan measurements (Figure 10). At higher input intensities, ESA dominates, effectively clamping the transmitted output power. Additionally, the presence of AgNPs enhances the local electromagnetic field via SPR, which further amplifies the NLA of the composite system. The enhancement in the electromagnetic field is not attributed to direct energy transfer between AgNPs and the perylene dye. Instead, it is primarily associated with localized surface plasmon–induced field enhancement, which increases the effective excitation probability and excited-state population of the dye. This plasmonic effect can enhance nonlinear absorption without implying directional energy transfer [59]. The enhanced OL efficiency with increasing AgNP concentration can thus be attributed to the combined effects of plasmonic field enhancement, NLA, and scattering contributions from the metal NPs [60,61]. These results demonstrate that Ag@TAIPDI nanocomposites exhibit strong, concentration-dependent OL performance, confirming their potential as efficient materials for laser protection, optical switching, and photonic limiting applications.

## 4. Conclusions

In this work, the linear, nonlinear, and optical limiting properties of TAIPDI dye doped with AgNPs were systematically investigated under femtosecond laser excitation. The AgNPs were synthesized using a clean, surfactant-free pulsed laser ablation technique, which yielded stable, spherical nanoparticles with an average diameter of approximately 18 nm. Incorporation of AgNPs into the TAIPDI matrix significantly influenced both the linear and NLO responses of the dye. The UV–visible absorption spectra revealed a clear plasmonic coupling effect, while the fluorescence measurements demonstrated a pronounced enhancement in emission intensity with increasing AgNP concentration, attributed to metal-enhanced fluorescence via localized surface plasmon resonance. Z-scan analysis confirmed that the Ag@TAIPDI nanocomposites (Ag@TAIPDI hybrid systems) exhibited reverse saturable absorption behavior and a negative nonlinear refractive index, indicating strong third-order NLO effects. Both the nonlinear absorption coefficient *β* and nonlinear refractive index *n*_2_ increased with AgNP concentration, resulting in a substantial enhancement of the overall third-order susceptibility. Furthermore, the optical limiting threshold decreased markedly with higher AgNP concentration, demonstrating improved optical limiting efficiency due to enhanced nonlinear absorption and local field amplification. The results establish that AgNPs–TAIPDI dye hybrid systems possess superior third-order NLO responses and excellent OL performance, making them promising candidates for future photonic devices, optical switches, and laser protection applications.

## Figures and Tables

**Table 1:** Summary of the NLO properties of Ag@TAIPDI nanocomposites at different AgNP concentrations.

Samples	AgNPs Conc. (mg/L)	AgNPs Conc. × 10^−10^ (M)	*ꞵ* × 10^−9^ (cm/W)	*n*_2_ × 10^−15^ (cm^2^/W)	Re [χ3]× 10^−16^ (esu)	$Im\left[\chi^{\left(3\right)}\right]$ × 10^−13^ (esu)	$\chi^{\left(3\right)}$ × 10^−13^ (esu)
Ag@TAIPDI nanocomposites	0	0	0.58	2.34	3.05	2.22	2.22
	2.3	1.21	1.35	5.43	8.12	6.79	6.79
	3.37	1.77	1.62	6.43	8.53	6.4	6.4
	4.5	2.38	1.65	6.86	7.79	4.78	4.78
	5	2.63	1.7	7.43	8.83	5.4	5.4

## Data Availability

Data are contained within the article.
